# Supplementary material for: Complete Genomic Characterization and Identification of Saccharomycopsis phalluae sp. nov., a Novel Pathogen Causes Yellow Rot Disease on Phallus rubrovolvatus
Source: J Fungi (Basel). 2021 Aug 28;7(9):707. doi: 10.3390/jof7090707 (PMC8468998; doi:10.3390/jof7090707)
Supplement: Supplementary file 1 [file jof-07-00707-s001.zip › Supplementary/Supplementary Figure S1.pdf]

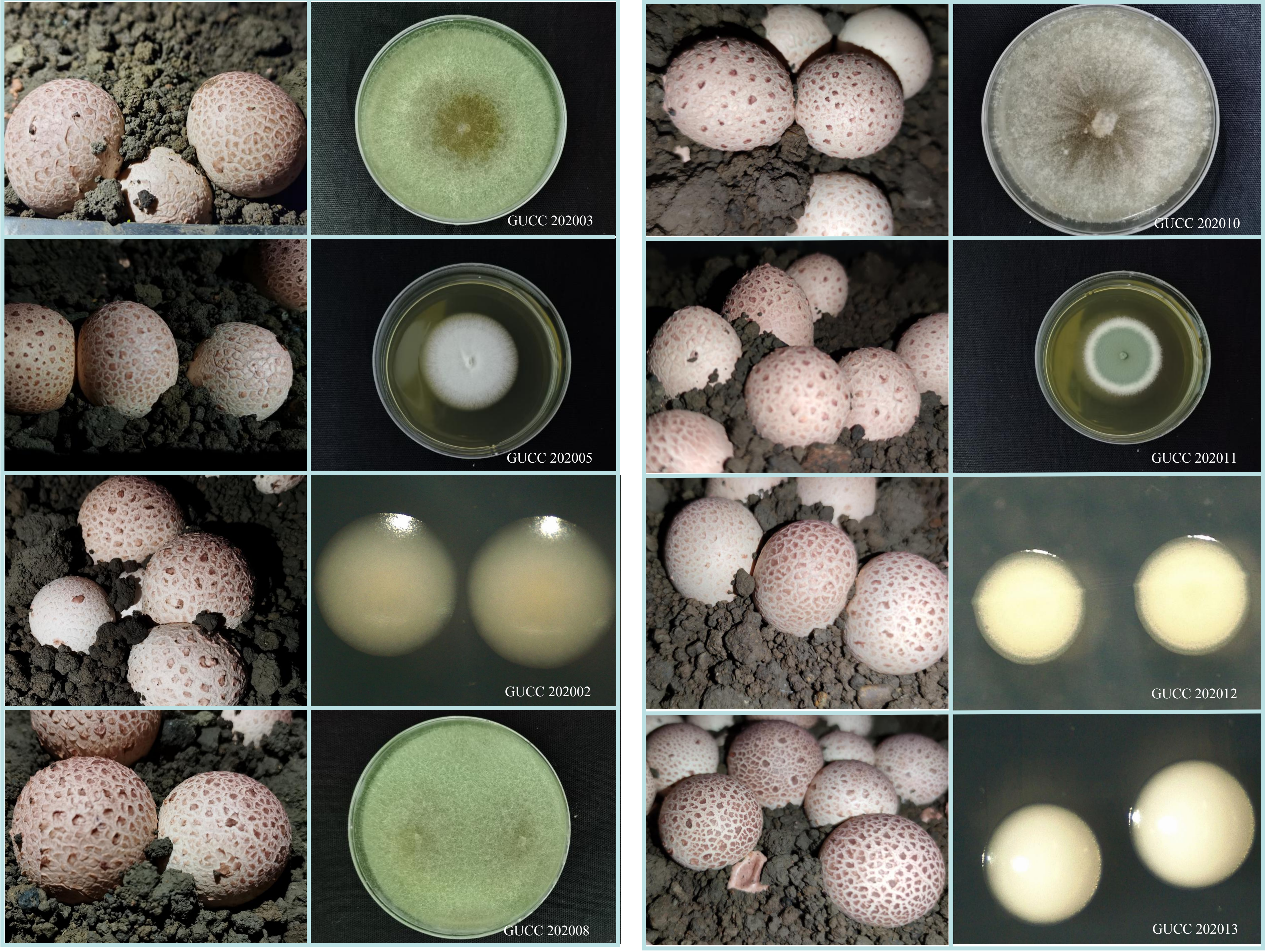
GUCC 202003
GUCC 202005
GUCC 202002
GUCC 202008
GUCC 202010
GUCC 202011
GUCC 202012
GUCC 202013

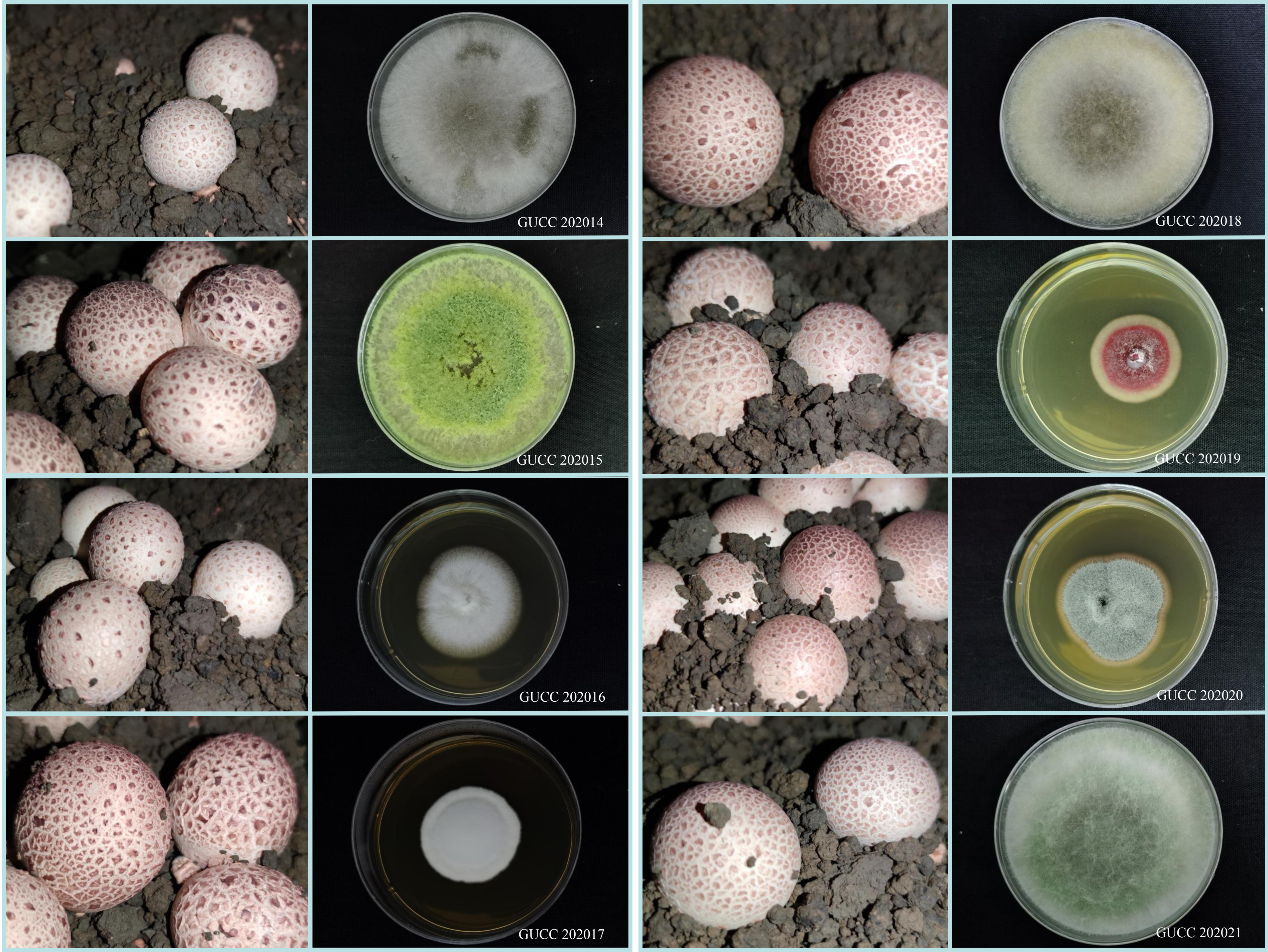
GUCC 202014
GUCC 202015
GUCC 202016
GUCC 202017
GUCC 202018
GUCC 202019
GUCC 202020
GUCC 202021

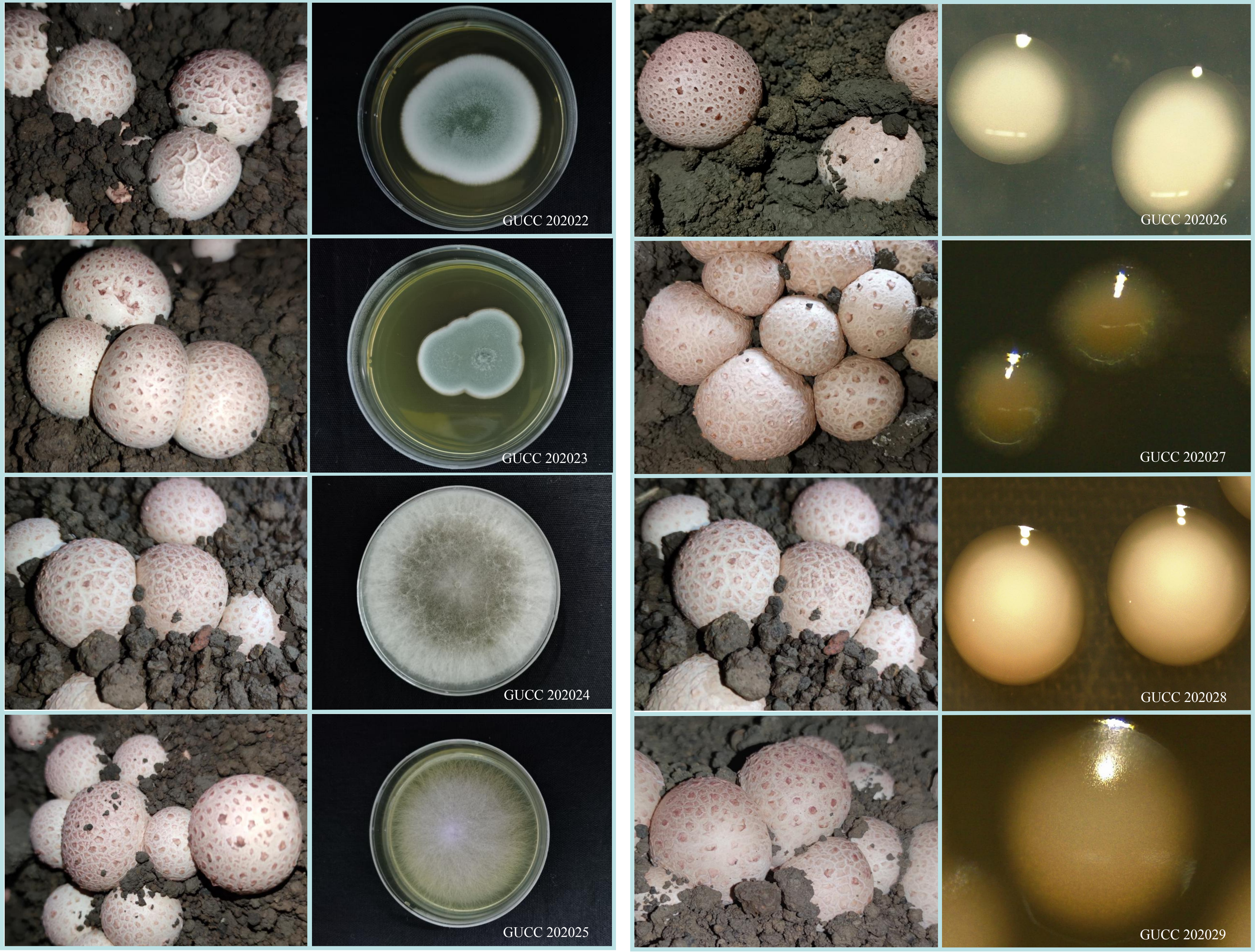
GUCC 202022
GUCC 202023
GUCC 202024
GUCC 202025
GUCC 202026
GUCC 202027
GUCC 202028
GUCC 202029

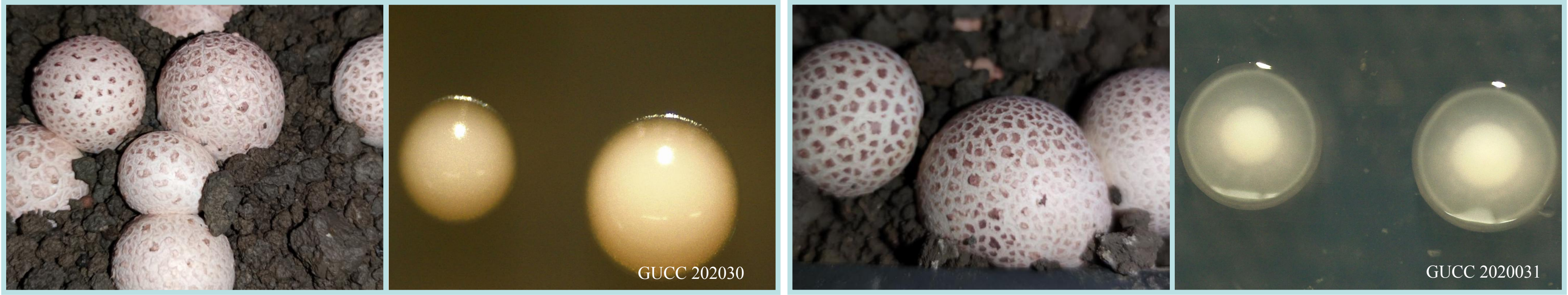
GUCC 202030
GUCC 2020031
